# Cybersecurity policy framework requirements for the establishment of highly interoperable and interconnected health data spaces

**DOI:** 10.3389/fmed.2024.1379852

**Published:** 2024-05-09

**Authors:** Christian Luidold, Christoph Jungbauer

**Affiliations:** Faculty of Computer Science, Multimedia Information Systems, University of Vienna, Vienna, Austria

**Keywords:** cybersecurity in healthcare, health data interoperability, risk management in health organizations, health data privacy, digital health ecosystems

## Abstract

This paper examines cybersecurity policy framework requirements for establishing highly interoperable and interconnected health data spaces, with a focus on the European Health Data Space (EHDS) and its corresponding joint action Toward European Health Data Space (TEHDAS). It explores the challenges of ensuring data security within an increasingly digital and collaborative healthcare environment, emphasizing the need for robust policy management to protect sensitive health information across diverse healthcare systems and supply chains. Through an analysis of use cases and held expert workshops, the study identifies key requirements for enhancing cybersecurity measures, fostering cross-border data exchange, and ensuring compliance with regulatory standards. It illustrates the practical implications of cybersecurity policies in a real-world scenario, demonstrating how they can be applied to enhance data security and policy effectiveness.

## 1 Introduction

In this paper, we analyze cybersecurity policy framework requirements for highly interoperable and interconnected health data spaces, with a focus on the European Health Data Space (EHDS) (EDHS)^1^ project “Toward European Health Data Space” (TEHDAS).^2^ We explore the significant challenges of securing data within an increasingly digital and collaborative healthcare environment. Our research leverages expert workshops and multiple use cases in a healthcare setting from the SPHINX project (1) to identify key requirements for enhancing cybersecurity measures, supporting cross-border data exchange, and ensuring compliance with regulatory standards. Each contribution is designed to offer actionable insights for policymakers and stakeholders in the healthcare sector.

### 1.1 Cybersecurity policy management at different levels

Effective policy management at all levels includes the development, implementation, monitoring, and enforcement of policies and best practices. This is extended by periodic assessments in order to ensure their relevance and validity supported by collaboration and communication between affected stakeholders. Below we briefly describe cybersecurity policy management at an organizational, interorganizational, and ecosystem level.

Organizational level: The main focus regarding policy management at an organizational level lies in the development and implementation of policies as guidelines pertaining to the organization's cybersecurity processes and practices in order to ensure compliance. Subjects included involve but are not limited to access control and incident response encompassing constant monitoring and enforcement of those policies.

Ecosystem level: Regarding policy management at an ecosystem level, the goal lies in the process of coordination of policies and practices between interconnected organizations in order to address shared risks. Main subjects include but are not limited to risks pertaining to the supply chain and third-party risk management, and involve a given degree of collaboration concerning the development and implementation of policies and practices.

Global level: Policy management at the global level focuses on the process of coordination of policies and practices between interdependent organizations within a broader scale encompassing entire sectors, e.g., critical infrastructures. Main subjects include but are not limited to information sharing and CTI in order to address cybersecurity risks. On the global level the collaboration involves parties from industry and government regarding the development and implementation of cybersecurity policies.

### 1.2 Aim and context of the research

The goal of our research work was to develop a data driven and risk-aware cybersecurity policy management framework for public organizations with an emphasis on health. The framework takes a systemic-holistic view on policy management, and is driven by organizational and user requirements, building on the integration of proven decision and organizational learning models with artificial intelligence concepts. Previous experience (especially during piloting and evaluation of the CS-AWARE project^3^) has shown that current approaches to policy management are not adequately addressing the dynamic nature of the cybersecurity environment (2) and requires further research in enhancing cybersecurity awareness, as well as in increasing the potential of interoperability of organizations beyond mere data exchange (3).

The dynamic nature of cybersecurity is already challenging on an operational level. It becomes increasingly unmanageable at the policy level, especially for public sector organizations and institutions that handle personal and sensitive data as it is the case of health service providers, hospitals, clinical research and care centers, etc. The need to quickly and dynamically adapt cybersecurity management policies (e.g., relating to risk management and business continuity, incident management) to keep up with the continuously changing threat and attack landscape requires a new and more dynamic approach to policy definition and constant re-evaluation against the requirements defined by the cybersecurity realities, as is reported by threat intelligence provided by, e.g., NIS competent authorities/CSIRTs or threat intelligence communities.

The proposed policy management framework will cover:

1. Support for policy requirement assessment and definition, based on the individual socio-technical requirements of organizations. This will be based on the socio-technical soft systems analysis conducted during the CS-AWARE-NEXT project.^4^
2. A dynamic and data driven continuous re-assessment of policy requirements using AI to dynamically reassess cybersecurity policies through continuous data-driven analysis. By integrating Argyris' double loop learning model (4), it allows for adaptive policy execution and adjustments based on evolving threats, with the inner loop focusing on execution and the outer loop on policy modification itself.
3. A decision support and management model that aids organizations in efficiently implementing and dynamically adjusting policies during cybersecurity incidents. It integrates the OODA Loop–Observe, Orient, Decide, Act–a model (5) suited for rapid and informed decision-making in dynamic environments. It informs adjustments and decision-making by monitoring threat intelligence and internal systems, analyzing risks, and ensuring the explainability of actions through contextualization.

The goal is to evaluate the potential for tighter integration of the dynamic operational cybersecurity management capabilities that CS-AWARE already provides with the organizational component that is defined by the policies. The piloting evaluation of the CS-AWARE project has shown that there is great potential in streamlining those two aspects, which requires a more dynamic approach to policy management.

This paper focuses on the requirements analysis regarding the development of risk-aware cybersecurity policy management. It builds upon the results of the conducted end-user workshops with pilot partners from Larissa in Greece. This section continues with the motivation and relevance pertaining to risk-aware cybersecurity policy management and a brief description of the classification of its usage at different levels. Section 2 we present a state-of-the-art analysis focusing on current trends and advances from a legal standpoint comprising of current standards and guidelines, followed by initiatives from affecting effective policy management. Additionally, key scientific work pertaining to planned design decisions is presented in more detail. Section 3 examines current challenges of implementing effective policy management from the points of transparency, information sharing, and responsibility and accountability. Section 4 presents the main key requirement specification from the results of the end-user workshops, followed by the conclusions in Section 5.

## 2 Current trends and advances in risk-aware cybersecurity policy management

Current trends regarding cybersecurity policy management are heavily influenced by the effects of legal frameworks including regulations, standards, directives, and laws. Given a shift toward increasingly placing responsibilities on the individual organizations, particularly the senior management, an increased presence of cybersecurity measures can be noted. A general trend is the usage of machine learning and artificial intelligence to implement and support new and existing cybersecurity measures. Following is a non-exhaustive description of current trends and advances:

- **Proactive risk management** is aimed at preventing cyber attacks before they occur instead of merely responding to them and their fallout as they arise. The main approach lies in implementing effective policies and risk management strategies.
- The **focus on risk assessment** forms a crucial component for effective cybersecurity policy management by supporting organizations to identify potential vulnerabilities and facilitating decision making concerning the prioritization of asset security.
- The **automation of policy management** streamlines the process of creating, implementing, and enforcing policies. By adding processes to include monitoring and maintenance during execution, freed resources can effectively be used to focus on more complex challenges.
- The **integration of threat intelligence** alleviates the efforts of organizations to stay ahead of emerging threats and facilitates timely responses to security incidents. Threat intelligence comprise collections of data and results of corresponding analyses about security incidents and vulnerabilities from various sources shared on various levels by entities including government agencies, CSIRTs, organizations, and communities.
- **Collaboration and information sharing** constitutes a driving factor regarding enhanced cybersecurity resilience and timely response to security incidents. It includes various organizations, governmental bodies, CSIRTs and communities working together involving sharing best practices, collaborating on issues, and coordinating actions pertained to security incidents.

Incorporating the above trends and advances into cybersecurity policy management can help to enhance the resilience of organizations by supporting the protection of assets and increase the preparedness against emerging cybersecurity threats.

### 2.1 Standards and guidelines

The requirements for CS-AWARE-NEXT are in part heavily influenced by current standards and guidelines with the most prominent being the GDPR, NIS2, ISO27001, and the NIST CSF. The following describes the fundamental aspects of each instance relevant to this project:

Starting with the GDPR (titled “General Data Protection Regulation”),^5^ the focus in the context of this document lies in the handling and processing of data by controllers and the associated rights of data subjects.

- **Right to data portability (Art. 20 GDPR)** states that the data subject shall have the right to receive data concerning themselves provided to a controller and transmit the data to another controller in a machine-readable way. The processing has to be carried out in an automated way.
- **Representatives of controllers or processors not established in the Union (Art. 27 GDPR)** states that the controllers or processors need to designate in writing a representative in the European Union, more precisely in a member state, where the data subjects, whose personal data are processed. The obligation of having a designated representative does not apply to public authorities or bodies.
- **Processing under the authority of the controller or processor (Art. 29 GDPR)** states that the processor and any person acting under the authority of the controller or of the processor having access to personal data shall not process those data except on instructions from the controller, unless required to do so by Union or Member State law.
- **Security of processing (Art 32 GDPR)**, specifically Art 32(2) states that in assessing the appropriate level of security account shall be taken in particular of the risks that are presented by processing, in particular from accidental or unlawful destruction, loss, alteration, unauthorized disclosure of, or access to personal data transmitted, stored or otherwise processed.

The NIS2 Directive^6^ (titled “Directive on measures for a high common level of cybersecurity across the Union”) is an EU-wide legislation on cybersecurity focusing on active risk management. It expands the scope of the original NIS Directive from 8 to 16 sectors and removes the threshold for applicability of the directive regarding the size of an organization of its corresponding sector. Furthermore, it requires improved risk management approaches, more stringent reporting obligations, harmonized sanctions, and enhanced cooperation with authorities and CSIRTs.

The ISO/IEC 27001:2022 (titled “Information security, cybersecurity and privacy protection - Information security management systems - Requirements”) is a European standard pertaining to IT security and management systems. It specifies the requirements for establishing, implementing, maintaining and continually improving an information security management system within an organizational context. These requirements are generic and intended to be applicable to all organizations, regardless of type, size, or nature. The corresponding ISO/IEC 27002:2022 (titled “Information security, cybersecurity and privacy protection - Information security controls”) standard^7^ provides a reference set of generic IT security controls including implementation guidance, which can be used for the development of organization-specific information security management guidelines, as well as implementing information security controls according to best practices. These standards are relevant to risk-aware cybersecurity management as they provide a comprehensive framework for managing cybersecurity risks.

The Cybersecurity Framework by the National Institute of Standards and Technology (NIST CSF)^8^ provides initial guidelines for improving cybersecurity risk management in critical infrastructures, pointing out its relevance to risk-aware cybersecurity management. It includes five framework functions as its core structure: Identify, Protect, Detect, Respond, and Recover. With the update to CSF2.0,^9^ scheduled for Winter 2024, the framework is set to provide a more extensive guidance regarding implementation, including more specific information about definitions, applications, and interoperability. Additionally, a new theme to be included is the consideration of cybersecurity risks in supply chains in the CSF.

### 2.2 Initiatives from industry, government, and professional organizations

Current initiatives regarding risk-aware cybersecurity policy management can be found from industry, government, and professional organizations. Their goal lies in increasing the resilience against cyberattacks and raising awareness concerning the presently changing threat landscape, as well as best practices, standards and regulations. Initiatives focus on various aspects of risk-aware cybersecurity policy management, including risk assessment, policy development and enforcement, collaboration, information sharing, and decision-making processes.

Prominent examples of initiatives from industry include the NIST Cybersecurity Framework for helping organizations to better understand and improve their management of cybersecurity risks. While it was originally developed for critical infrastructures, many countries across the globe have adopted and adapted the Cybersecurity Framework with some considering its use as mandatory for both private and public sector.

Regarding initiatives from governments, the European Union's GDPR and NIS2 (the latter specifically targeting critical infrastructures) have caused a significant impact on managing cybersecurity risks. They include provisions for data protection and cybersecurity, as well as requiring organizations to implement technical and organizational measures to ensure an appropriate standard concerning cybersecurity.

Initiatives from professional organizations commonly include education material and certification programs in the domain of cybersecurity. Organizations like the Cloud Security Alliance (CSA)^10^ additionally provide publications and documents on latest research conducted in the field of cloud security, as well as providing networking opportunities for members.

The latest pivotal initiative in the context of this paper is the EHDS by the EU. The proposition aims for granting natural persons a higher degree of control over their electronic health data. By ensuring a common legal framework across the EU it would enhance the quality of healthcare-related services, as well as creating a single market with agglomerated healthcare data made available in a preprocessed format for researchers, innovators, and policy-makers. This shall be achieved through establishing strong cybersecurity measures focused on the aspect of data exchange within a highly interoperable environment. In conjunction with TEHDAS the new proposition also focuses on an increased stakeholder engagement encompassing different roles and expertise, as well as to support the process of collaboratively developing and implementing effective policies including cybersecurity policies.

### 2.3 Scientific works

Main considerations pertaining to design decisions and their implementation of a risk-aware cybersecurity policy management framework within this WP are taken from established methods. Taking the standards and guidelines, as well as the current initiatives from the previous subsections into account, the key scientific works is composed of the following research:

The core concept of effective policy management including the performance monitoring of individual policies is defined by the double-loop learning model developed by C. Argyris, which can be applied to a variety of contexts, including education, personal growth and development, and organizational change. It describes a learning process in which individuals and organizations critically examine and question the underlying assumptions and values regarding their actions and decisions. The results of an effective implementation can lead to more efficient and lasting learning and growth effects. In the context of this research two main types of learning were identified: single-loop learning and double-loop learning. Single-loop learning occurs when an individual or organization works to correct or improve actions or outcomes without questioning the underlying assumptions and values regarding their behavior. It is focused on solving problems instead of exploring the causes of those problems. Double-loop learning involves a deeper level of analysis and questioning by requiring individuals and organizations to critically examine their assumptions and values pertaining to their actions and decisions and questioning their validity and appropriateness. Beyond just involving actions regarding correction or improvement, its view also involves questioning and potentially changing the fundamental values (4).

Research conducted by J. Boyd explores mental patterns or concepts of meaning pertaining to individuals to shape and be shaped by a changing environment. The identified basic goal of everyone lies in improving the capacity for independent action. Any level of cooperation or competition exists to satisfy this aim. If a desired level of independence cannot be achieved, compromises are taken, and constraints are developed in order to collectively pool skills and talents to overcome or remove obstacles. If overcoming or removing still proves to be impossible the group might alienate and lose members for whom these hindrances are deemed important. In order to strengthen alliances pursuing their goals, effective decisions have to be taken and resulting actions are to be monitored. This creates a need for decision models developed for constantly changing environments. Before new models can be implemented, existing models or concepts, which might inhibit the new one need to be separated from the rest of its associated domain and unstructured by a mental concept coined as “destructive deduction”. The subsequent restructuring and creation of new models or concepts by piecing together individual bits to conform to given needs was coined “creative induction”. The relation and application of these mental concepts are employed to formulate decision models for individuals and groups to determine and monitor actions to address incidents in changing environments and therefore improve their capacity for independent actions (6).

The OODA (Observation-Orientation-Decision-Action) loop introduced by Boyd resulted from the effort to describe the nature of adversarial engagements. OODA time cycle or loop suggests that success in war depends on the ability to out-pace and out-think the opponent, or put differently, on the ability to go through the OODA cycle more rapidly than the opponent. In cybersecurity the process allows stakeholders to learn from previous experiences, feeding lessons learned into the loop activities to achieve better performance contains four steps. Each group of stakeholders must make observations and process those observations through the orientation process, then use orientation in the decision process, then turn the decisions into actions, which in turn change the world being observed. The focus of the OODA loop is not about making faster decisions, but rather about manipulating the environment to “inhibit an adversaries capacity to adapt to such an environment (suppress or distort observations)”. The environment is seen as a means of disorientation to disrupt the adversary's decision-making. Rather than operating in isolation, decision and execution cycles take place simultaneously, but not in synchronization, for both sides. The conflict in the minds of the adversaries compromises the cognitive dimension of the information environment. Adding the cognitive dimension to cyberspace changes the analysis of cyberspace operations from a search for vulnerabilities in hardware and software into an engagement including information operations. “Situational awareness” is a term from psychology which describes both a field of study and the coupling of actors to their operating environment. Situational awareness is knowing what's going on around you (7).

## 3 Special challenges of risk-aware cybersecurity policy management in interdependent health organizations

An important aspect of the EHDS is risk management, as the proposal was specifically designed to take the NIS Directive into account to include measures to mitigate identified risks. Risk management typically focuses on credit risk, market risk, and operations risk. Technology risk constitutes a subset of operations risk, and cybersecurity risk subsequently is a part of technology risk. Given the fact that cybersecurity risk would generally be found on the lower end of the risk hierarchy it is often absent from centralized risk management processes. Despite focusing on technological risks stemming from software, the predominant driving factor for risks in operation is human error. Software engineers more commonly tend to exercise their authority to bypass software restrictions and therefore inhibit developed security measures.

Cybersecurity constitutes a crucial challenge for the health sector since it influences the security, privacy, and quality of the provision of healthcare services, especially in interconnected systems and services, as aimed by the EHDS. Nonetheless, handling cybersecurity risks in interdependent healthcare organizations presents several challenges, which arise from the intricacy, heterogeneity, interconnectivity, dynamics, and resource limitations of the sector. Therefore, a comprehensive and collaborative approach is essential for developing a risk-aware policy management framework, enabling healthcare organizations the identification, assessment, prioritization, and mitigation of cybersecurity risks while considering security and usability requirements. It is crucial to involve all stakeholders and align the framework with industry standards and best practices. Furthermore, the cybersecurity framework ought to possess adaptability and flexibility to effectively manage the dynamic and evolving cyber threats faced by the healthcare industry, while catering to the sector's increasing needs and expectations.

### 3.1 Key challenges in health organizations

Information security risk assessment focuses on the potential damage to data subjects regarding the confidentiality, integrity, and availability of data. The integration of new security measures is generally decided upon calculating the expected loss through the sustained damage taken and comparing it to the cost of implementation. Problems arise by nature of not knowing the actual performance of those security measures, making the quantification of costs an issue.

Risk assessment as a management tool should be distinguished between risk management and security management. Risk management encompasses strategies involved in decision-making and the subsequent monitoring of the outcomes. Security management encompasses programs, processes, etc. used according to the decisions made from the risk management. Risk management therefore constitutes the integral part for cybersecurity policies and cybersecurity policy management (8).

The most prominent issues pertaining risk management focus on the organizational responsibility to assess risks, individual responsibilities or segregation of duties and the role of the government regarding the assurance of effective risk management practices. Specifically, the shift regarding the placement of responsibility on senior management governed the last years, predominantly through the GDPR, as well as NIS and the upcoming NIS2. This shift was taken into account in defining the proposition of the EDHS in the context of including a broader spectrum of stakeholders, especially regarding policy development and project management.

Managing cybersecurity policies in interdependent health organizations can present unique challenges due to the complex relationships and dependencies that exist between these organizations. Listed below is an overview of special challenges determined during the end-user workshops which can arise in this context:

**Varying levels of cybersecurity maturity:** Interdependent health organizations may have different levels of cybersecurity maturity and understanding, which can make it difficult to coordinate policies and practices effectively. The difference between small local companies and large organizations might be very large, which can make it challenging to establish a common set of policies and standards.

**Limited resources:** Small local health organizations may have limited resources to allocate to cybersecurity policy management, which can make it challenging to implement and enforce policies effectively. This can be particularly challenging for smaller healthcare institutions that may not have dedicated cybersecurity staff or budgets.

**Complex interdependencies:** Different regional organizations may have complex interdependencies that can make it challenging to coordinate policies and practices. For example, a regional healthcare system may rely on multiple local clinics and hospitals to provide patient care, which can make it challenging to establish common cybersecurity policies and practices across the entire system.

**Regulatory and compliance requirements:** Health organizations may be subject to different regulatory and compliance requirements, which can make it challenging to establish a common set of cybersecurity policies and practices. For example, hospitals are subject to different data protection regulations than organizations in the food industry, which can make it challenging to establish common policies related to data protection.

**Communication and coordination challenges:** Interdependent health organizations may face communication and coordination challenges when trying to establish common cybersecurity policies and practices. This can be particularly challenging when organizations have different priorities or when there is limited communication and collaboration between stakeholders.

Overall, managing cybersecurity policies in interdependent local and regional organizations requires a collaborative and coordinated approach that takes into account the unique challenges and dependencies that exist between these organizations. This may involve establishing common policies and standards, sharing information and resources, and investing in cybersecurity training and education for staff.

### 3.2 A comprehensive scenario for secure digital healthcare

The European Health Data Space (EHDS) initiative, implemented by the European Commission, aims to facilitate secure and ethical utilization of health data throughout the EU. The EHDS is designed to improve the quality and efficiency of healthcare services, while promoting research and innovation in the health sector. However, the implementation of the EHDS poses challenges for interdependent healthcare organizations in terms of risk-conscious cybersecurity policy management. In order to demonstrate the importance of cybersecurity management a comprehensive scenario in a healthcare setting was created combining 4 use cases from the Horizon 2020 project SPHINX (1). The scenario combines the following use cases:

1. UC13: Exploiting Remote Patient Monitoring Services,
2. UC24: Theft of Patient Data using the Telemedicine System,
3. UC17: Accessing Health Data from a Fitness Tracker, and
4. UC20: Compromised Workstation Allows the Scanning of Hospital Network.

The complex scenario depicts a combination of exploitation of remote patient monitoring services and vulnerabilities in telemedicine systems leading to unauthorized access of health data, including data from fitness trackers. In conjunction with compromised workstations the scenario evolves into a multi-faceted cyber threat illustrating the dynamics of cybersecurity in healthcare, with a particular focus on emerging technologies and remote healthcare delivery. The unified scenario balances patient monitoring and data management together with cybersecurity measures to represent a necessary standard for integrating technology and security to enhance patient care and privacy.

The following subsections give an overview of the individual use cases followed by an analysis of included issues and proposed relevant cybersecurity policies.

#### 3.2.1 UC13: exploiting remote patient monitoring services

Using a remote patient monitoring service, a patient uses a mobile App to read vital signs captured by medical devices and upload the unencrypted data via a home Wi-Fi router. By cracking the weak password and forcing communications to non-transport layer security (TLS) mode a hacker was able to modify health-related information sent to the server. This resulted in the attacker compromising the trust and data integrity of the provided medical services, creating false alarms and causing emergency actions from the personnel monitoring the patient. An analysis of the relevant policies is depicted in Table 1.

**Table 1:** Analysis of policies in UC13.

**Policy area**	**Current state**	**Recommended policy**	**Policy management action**	**Expected outcome**
Encryption standards	Patient vital signs data not sent encrypted	Mandatory use of encryption for all data transmissions	Regular security audits to ensure encryption implementation	Enhanced security of patient data transmission
Network access control	Home WiFi router protected by a weak password	Strong password policy for home WiFi router	Implement password strength and complexity checks	Prevention of unauthorized network access
Device authentication	Mobile app connects to the Internet via home WiFi router	Mobile app must authenticate the remote patient monitoring platform before uploading data	Firmware update to enforce platform authentication	Reduction in the risk of man-in-the-middle attacks
Data integrity	Lack of verification of data received by the remote patient monitoring platform	Implementation of data integrity checks	Continuous monitoring for data anomalies	Assurance of accurate patient vital signs data

#### 3.2.2 UC24: theft of patient data using the telemedicine system

By exploiting a Web Real Time Communication (WebRTC) bug in a hospitals telemedicine service, an attacker was able to stealthily connect to an active media session between a patient and their doctor using a Man-in-the-Middle (MitM) attack. With this the hacker was not only able to access the audio and video stream of the session but could also access and compromise the patient's Electronic Medical Record (EMR) data. The attacker also introduced a crypto-ransomware into the hospital's network, threatening to destroy patient data. This resulted in the loss of availability of healthcare databases, impacting or preventing IT-based healthcare services for up to 2 months and compromising the trust of patients into the healthcare organization due to violating the confidentiality, integrity, and availability of the patient's data. An analysis of the relevant policies is depicted in Table 2.

**Table 2:** Analysis of policies in UC24.

**Policy area**	**Current state**	**Recommended policy**	**Policy management action**	**Expected outcome**
Encryption standards	WebRTC bug leaking the customer's IP address	Mandatory use of WebRTC security features	Regular security audits to ensure WebRTC security	Enhanced privacy of patient communication
Network access control	Compromised signaling server	Restricted access to signaling server	Implement network monitoring and access logs	Prevention of unauthorized network access
Device aAuthentication	Lack of verification of peer connection	Implementation of peer identity verification	Firmware update to enforce peer identity verification	Reduction in the risk of man-in-the-middle attacks
Data integrity	Lack of verification of data sent to EMR	Implementation of data integrity checks	Continuous monitoring for data anomalies	Assurance of accurate patient EMR data

#### 3.2.3 UC17: accessing health data from a fitness tracker

An orthopedic center recommends the usage of GNSS-enabled fitness trackers for improving the quality of patient diagnosis by connecting to the centre's WiFi and server. A hacktivist replicates the centre's WiFi SSID and subsequently launches a man-in-the-middle attack, intercepting and manipulating patient data transmitted to the server, as the used encryption was based on a known symmetric algorithm utilizing plain HTTP without TLS. The tampered data registering on the centre's real network server raises alarms among the medical staff, therefore binding additional resources. This attack resulted in the violation of confidentiality and integrity of patient data impacting the centre's quality of services and subsequently the patient's private life, which consequently eroded the centre's credibility. An analysis of the relevant policies is depicted in Table 3.

**Table 3:** Analysis of policies in UC17.

**Policy area**	**Current state**	**Recommended policy**	**Policy management action**	**Expected outcome**
Encryption standards	Use of known symmetric encryption without TLS	Mandatory use of TLS for all communications	Regular security audits to ensure TLS implementation	Enhanced security of patient data transmission
Network access control	Unrestricted WiFi access	Restricted WiFi access with authentication	Implement network monitoring and access logs	Prevention of unauthorized network access
Device authentication	Fitness trackers connecting to any network SSID	Devices must authenticate the network before connecting	Firmware update to enforce network authentication	Reduction in the risk of man-in-the-middle attacks
Data integrity	Lack of verification of data sent to server	Implementation of data integrity checks	Continuous monitoring for data anomalies	Assurance of accurate patient health data
Patient privacy	Potential for patient data and location access	Strict access controls for sensitive data	Training staff on privacy policies and procedures	Protection of patient's private information

#### 3.2.4 UC20: compomised workstation allows the scanning of hospital network

By opening an attachment of an email containing a trojan, an employee causes the compromise of a hospital workstation by a hacker, who establishes a backdoor to launch a network scanner. This allows the hacker to gather detailed information about the hospital's IT assets, as well as information about operating systems, browsers, and network protocols in order to exploit vulnerabilities and strengthen the attacker's presence. This access can subsequently be used to impact IT-dependent healthcare services or compromise the confidentiality, integrity, and availability of patient data. An analysis of the relevant policies is depicted in Table 4.

**Table 4:** Analysis of policies in UC20.

**Policy area**	**Current state**	**Recommended policy**	**Policy management action**	**Expected outcome**
Email security	Employee opening an email containing a Trojan	Implementation of email filtering and scanning	Regular security training and awareness for employees	Prevention of malware infection via email
Asset management	Lack of information about the hospital's IT assets	Implementation of asset inventory and classification	Continuous monitoring and updating of asset information	Improved visibility and control of IT assets
Data protection	Potential for patient data access, modification, or disclosure by the hacker	Implementation of data encryption, backup, and recovery	Continuous monitoring and reporting of data breaches	Assurance of patient data confidentiality, integrity, and availability

### 3.3 Cybersecurity policy management and transparency

One of the key challenges in cybersecurity policy management is balancing the need for transparency with the need to protect sensitive information. Reluctance to disclose details about cybersecurity policies and practices for fear of revealing exploitable vulnerabilities is common, which caused a lack of standardized reporting for cybersecurity policy management until legal frameworks took effect. Despite these recent changes, a significant number of organizations struggle to understand and implement guidelines for reporting. As the threat landscape is constantly changing, keeping cybersecurity policies and best practices up-to-date can be challenging.

Many organizations are also subject to regulatory requirements related to cybersecurity, which can create challenges in managing policies and practices. A lack of awareness among stakeholders about the importance of cybersecurity policy management and the risks associated with cyber-attacks can further create barriers to enhance organizational resilience. Addressing these challenges through awareness trainings, dedicated resources, and enforced policies has a significant impact on an organization's cybersecurity resilience and facilitates compliance with legal regulations (8, 9).

### 3.4 Sharing cybersecurity policy management approaches in interdependent organizations

Sharing cybersecurity policy management approaches as a form of collaboration between interdependent organizations facilitates understanding of risks and risk management, including the identification of areas of concern, aiming at establishing a common baseline regarding policies and practices. One of the key challenges to achieve this objective lies in the heterogeneity of organizations. Differences in organizational structures mean differences in risk strategies and tolerances, which inhibit the development of shared policies and practices.

Another aspect is defined through used infrastructure and technology. Organizations relying on cloud services will have corresponding policies which differ from those organizations utilizing on-premise infrastructure. Paired with different priorities pertaining to individual sectors (e.g., water supply vs. healthcare) establishing a common focus can be difficult.

An additional challenge lies in regulations and legal constraints. A lack of trust constitutes the inhibiting factor with regard to sharing cybersecurity policy management approaches, predominantly when it comes to sharing sensitive information. Organizations competing in the same industry might further exhibit reluctance in sharing approaches presenting additional barriers for collaboration.

Addressing these challenges through established guidelines for sharing information and dedicated communication channels facilitates the alignment of policies and practices. Furthermore, trust can be built through regular communication and collaboration activities supporting decision making and enhancing cybersecurity resilience of participating organizations (9, 10)

### 3.5 Responsibility and accountability for cybersecurity policy management

Cybersecurity policy management encompasses a significant amount regarding challenges related to responsibility and accountability as it constitutes a shared responsibility involving multiple stakeholders across an organization. One of the challenges is the lack of clear ownership for cybersecurity policies, which complicates holding individuals or groups accountable for breaches or failures. Another challenge is the existence of blame culture involving individuals or groups being blamed for cybersecurity incidents rather than focusing on addressing the root causes of the incident resulting in the creation of a hostile environment discouraging collaboration and information sharing, further inhibiting efforts to enhance cybersecurity resilience. Furthermore, effective cybersecurity policy management can be resource-intensive, requiring significant investments in technology, training, and personnel. Limited resources combined with issues pertaining to ownership impede the allocation of responsibility and accountability.

Due to the evolving cybersecurity threat landscape effective cybersecurity policy management requires monitoring and maintenance of policies and practices including aspects regarding responsibility and accountability. This is often triggered by changes in compliance and regulatory requirements (e.g., NIS2) affecting cybersecurity policies and practices, possibly creating additional responsibilities and accountabilities pertaining to policy management. Addressing these challenges through establishing clear ownership including a culture of collaboration and information sharing, as well as allocating resources to cybersecurity and actively maintaining cybersecurity policies creates an important baseline for strengthening an organizations cybersecurity posture. Legal compliances and regulations provide goals for implementing clear processes for reporting and investigating cybersecurity incidents further inhibiting the effects of blame culture and facilitating the establishment of a resilient cybersecurity culture (9, 11).

## 4 Use case

**Figure 1:**
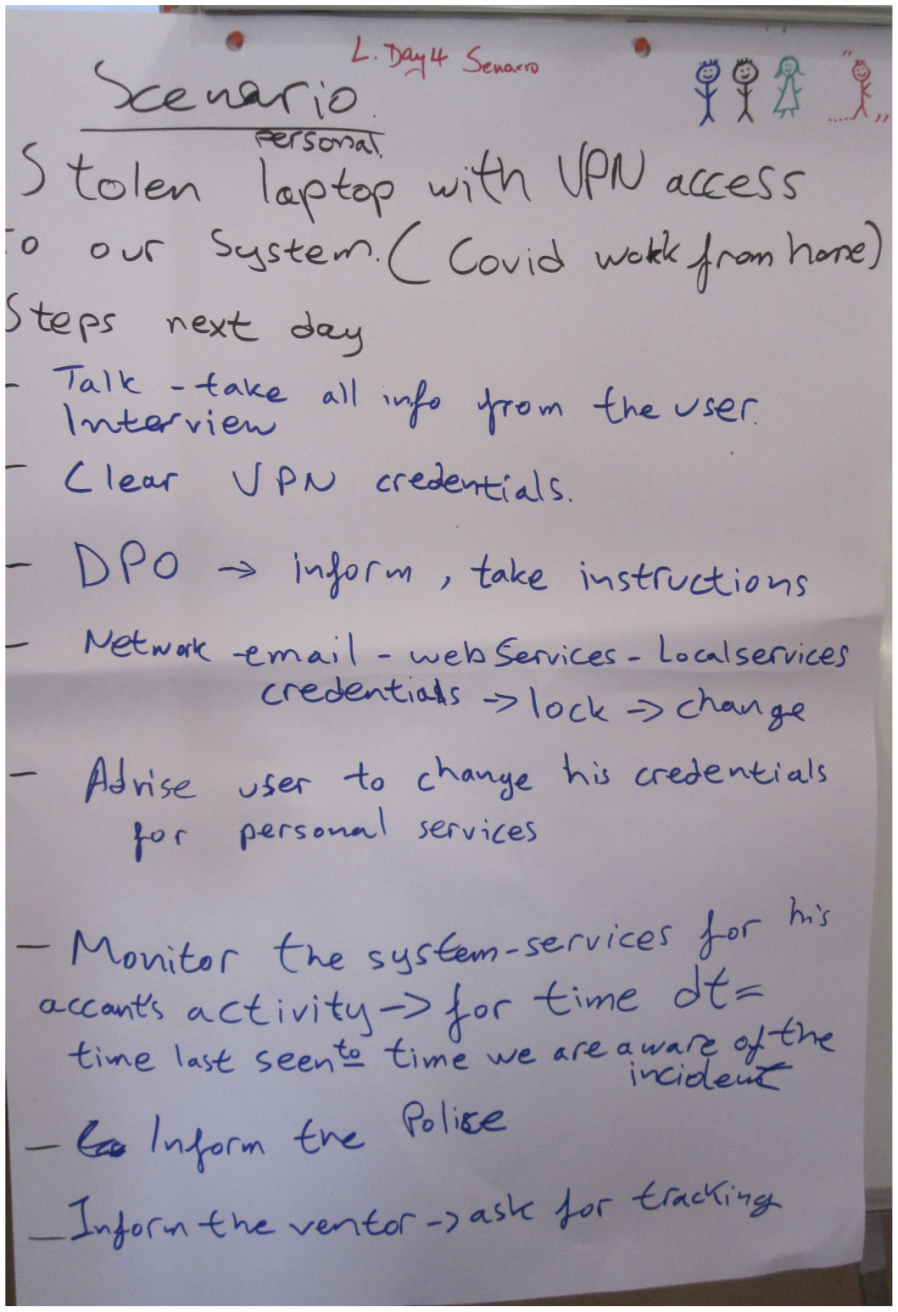
Original scenario from the workshop: stolen IT property.

A Use Case based from the CS-AWARE-NEXT project is used to prove the applicability in a real life scenario. The Case handles the response to a stolen Laptop with VPN Access as shown in Figure 1. This use case illustrates the practical implementation and challenges of the cybersecurity strategies and frameworks discussed in Section 3.2. By exploring a real-world scenario, we highlight the need for adaptable and robust cybersecurity measures to effectively address emerging threats, and demonstrate the direct application of risk-conscious cybersecurity policy management in a dynamic healthcare environment.

**Scenario** In the wake of increased remote work due to the COVID-19 pandemic, a laptop belonging to an employee has been reported stolen. This device has established VPN credentials, providing potential unauthorized access to the organization's secure network.

**Actors** User (employee from whom the laptop was stolen), IT Security Team, Data Protection Officer (DPO), Network Services Team, Police, Vendor (laptop provider)

**Preconditions** The employee has been working remotely due to pandemic restrictions and has been using a VPN to access the company's network. The laptop is equipped with the company's standard security features, including VPN access.

**Trigger** The theft of the laptop is reported by the user to the IT Security Team.

**Narrative**

- Upon receiving the report of the stolen laptop, the IT Security Team initiates an interview with the user to gather comprehensive information about the incident and the potential data at risk. The team works swiftly to clear the VPN credentials associated with the stolen device to prevent any unauthorized access to the network.
- Simultaneously, the Data Protection Officer is informed of the breach, and instructions are taken to comply with data protection laws and regulations. The DPO initiates the process of legal and notification obligations, including communication with law enforcement.
- Simultaneously, before it is known which data could be accessed through the device, the local authorities, banks, the CSIRTs and internal affairs need to be informed. The DPO is contacted regarding legal guidelines, as well as the manager and the national application/ internet provider.
- The Network Services Team jumps into action, conducting an immediate audit of all associated network, email, web, and local services credentials linked to the user's account, as well as personal data stored on the device. They lock down access and initiate a change of all passwords and security protocols as a precautionary measure. The device in question gets completely disabled.
- While the technical teams address the network and system vulnerabilities, the user is advised to change their credentials for personal services that may have been saved or accessed through the stolen laptop, to prevent further personal risks.
- With security measures in place, monitoring is heightened to track any suspicious activity across the system services associated with the user's account. The period of activity from the last known legitimate login to the current time is reviewed to assess any unauthorized actions taken.
- In conjunction with the internal monitoring, the vendor from whom the laptop was sourced is notified, and assistance is requested in tracking the device, if possible, through any built-in location services or tracking technologies that may have been part of the laptop's security features.

**Outcome** The immediate and coordinated response effectively mitigates the risks associated with the stolen laptop. The company's actions prevent unauthorized access, protecting sensitive data and maintaining compliance with cybersecurity policies. The user is made aware of the steps taken and is educated on the importance of securing personal and professional data. All parties remain vigilant, ready to respond to any subsequent activities related to the incident.

**Postconditions** The IT Security Team, along with the DPO, reviews the incident to update and refine the organization's security protocols and training, with the aim of preventing similar breaches in the future. Additionally, a follow-up with law enforcement and the vendor is maintained to track the progress on the recovery of the stolen property.

## 5 Key requirements specification for cybersecurity policy management

A successful cybersecurity policy management framework includes a range of vital components, including risk assessment, policy development and enforcement, collaboration and information sharing, and effective decision-making processes. Furthermore, it requires the involvement of internal and external stakeholders, with the latter encompassing government agencies, as well as other organizations.

Adding to the general components, individual requirements from organizations need to be taken into account to ensure a limited degree of restrictions and facilitate the adoption of a cybersecurity policy management framework. The main requirement categories obtained from the end-user workshops were examined and subsets of requirements were defined.

### 5.1 Basic knowledge and understanding of formalized policies

“Facilitate the understanding of documented formal policies and their advantages.” This meta-requirement focuses on raising awareness of stakeholders through training. The involved approach (12) is based on the Erasmus+ project COLTRANE. The sub-requirements derived from this meta-requirement are listed below:

- **Raise awareness of current policies:** Improve dissemination of policies from pure publishing to awareness, understanding and enforcement.
- **Promotion of collaboration and awareness raising:** Build on the COLTRANE approach for promoting collaborative policy management and awareness raising.
- **Simulation and training:** Use a virtual platform to simulate the handling of attack situations. Provide hands-on experience of collaboration- and awareness-driven policy management.
- **Organizational prerequisites for acting on the ecosystem level:** in order to handle policies at the ecosystem level organizations need to provide the necessary basis. The steps toward it have to be identified.

### 5.2 Formalization of best practices

“The ability to create documentation of best practices & guidelines in the organization to retain expertise and prevent loss of knowledge.” This meta-requirement focuses on the collaborative approach involving employees, organizations, communities, and government agencies in order to enhance an organization's resilience. In order to implement effective formalization of best practices, the cybersecurity policy management component works in concert with the collaboration component in WP2. An overview is listed below:

- Definition of state of the art practices: Facilitate the creation and maintenance of practices depending on current situations.
- Effective applicability and adaptability: Ensure practices are case-type based to provide a best fit for specific environments.

### 5.3 Shared policy repository

“Enable information sharing through a shared repository.” This meta-requirement regarding the provision and usage of a shared knowledge base regarding CTI, reports, as well as information pertaining to legal compliance. In order to realize the requirements of a shared policy repository and its subsequent usage a cybersecurity policy management component demands the support of AI-based quality data assessment and correlation. An overview is listed below:

- Harmonization with governing bodies: Ensure effective collaboration with governing bodies through establishing a common standard for information sharing.
- Provision of information: Make related documents from communities and governing bodies available for improving legal and technical readiness.
- Filter information according to needs: Enable means of distinction between policies according to metadata.
- Highlight current threats and vulnerabilities: Point out trending topics within the organization, community, and governing bodies. Analyze shared enriched CTI.

### 5.4 Implementation of best practices into workflows

“Enable the adaption of policies and best practices to the needs of the organization and their subsequent adoption into the organizational context.” This meta-requirement focuses on the adoption of policies into automatic workflows regarding disaster recovery and business continuity plans, therefore enhancing resilience and supporting legal compliance. In order to effectively implement best practices into organizational workflows the cybersecurity policy management component needs to encompass functionality pertaining to business continuity and disaster recovery. An overview is listed below:

- Provision of core essentials: Ensure the basic needs of an organization are met for legal compliance with governing bodies.
- Policy management life cycle: Provide an environment for creating, managing, enforcing and maintaining policies.
- Adaption of disaster recovery and business continuity plans: Facilitate the integration of policies and best practices into disaster recovery and business continuity procedures. Enable continuous monitoring and adaption of related workflows.
- Provide a basis for decision making: Building on decision making and reflective learning models in support of policy enforcement and maintenance.

### 5.5 Ensure effective visualization

“Create a visualization supporting the implemented functionalities in an intuitive way.” In order to stimulate an active engagement with the cybersecurity policy management component, the user interface and user experience need to appeal to the end user's preferences.

## 6 Conclusion

The need to adapt cybersecurity management policies quickly and dynamically (e.g., relating to risk management and business continuity, incident management) to keep up with the continuously changing threat and attack landscape requires a new and more dynamic approach to policy definition and constant re-evaluation against the requirements defined by the cybersecurity realities, as is reported by threat intelligence provided by, e.g., NIS competent authorities/CSIRTs or threat intelligence communities.

One of the aspects of collaboration within a shared ecosystem lies in the development of common policies and standards in order to diminish the complexity regarding the management of cybersecurity risks and ensuring actions taken are streamlined according to the same security protocols. Additionally, the implementation of common policies and standards helps to build trust between interdependent organizations and their customers, further increasing the relevance of effective risk-aware cybersecurity policy management.

The main focus regarding policy management at an organizational level lies in the development and implementation of policies as guidelines pertaining to the organization's cybersecurity processes and practices in order to ensure compliance. Main subjects include but are not limited to risks regarding the supply chain and third-party risk management, and involve a given degree of collaboration concerning the development and implementation of policies and practices. Managing cybersecurity policies in interdependent local and regional organizations can present unique challenges due to the complex relationships and dependencies that exist between these organizations. Local and regional organizations may be subject to different regulatory and compliance requirements, which can make it challenging to establish a common set of cybersecurity policies and practices.

Sharing cybersecurity policy management approaches in interdependent organizations has to keep in mind the differences in organizational structure, which can make it challenging to align cybersecurity policies and practices across different organizations. Sharing these sensitive cybersecurity policy management approaches requires a high degree of trust between organizations, which can be difficult to establish and maintain. Ultimately, a shared approach to cybersecurity policy management can help to improve the overall security posture of interdependent organizations and reduce the risk of cyber attacks.

Ongoing research focuses on support for compliance with regulatory bodies and authorities, as well as autonomous adaption to organizational events based on log data. This approach focuses on the use of the double-loop learning model to change minor policy details automatically, or provide decision making support for more substantial changes. Additional focus lies in addressing the development of security and privacy related policies for IoT devices in healthcare. Frameworks targeting compliance with security standards before deployment serve an increased demand in light of legislative plans for fostering data exchange, collaboration, as well as supply chain security.

## Author contributions

CL: Writing—original draft, Writing—review & editing. CJ: Writing—original draft, Writing—review & editing.

## Footnotes

^1^ https://eur-lex.europa.eu/legal-content/EN/TXT/?uri=celex:52022PC0197

^2^ https://tehdas.eu/

^3^ https://cordis.europa.eu/project/id/740723

^4^ https://cordis.europa.eu/project/id/101069543

^5^ https://eur-lex.europa.eu/eli/reg/2016/679

^6^ http://data.europa.eu/eli/dir/2022/2555/oj/eng

^7^ https://www.iso.org/standard/82875.html

^8^ https://www.nist.gov/cyberframework

^9^ https://www.nist.gov/cyberframework/updating-nist-cybersecurity-framework-journey-csf-20

^10^ https://cloudsecurityalliance.org/

## References

[1] Manso M. Use Cases Definition and Pilot Overview Document v3. (2021). Available online at: https://zenodo.org/records/5052727 (accessed December 7, 2023).

[2] Schaberreiter T Wieser C Koumpis A Luidold C Andriessen J Cappiello C. Addressing critical issues and challenges for dynamic cybersecurity management in organisations and local/regional networks: the CS-AWARE-NEXT project. In: 2023 Fifth International Conference on Transdisciplinary AI (TransAI). (2023). p. 232–236. Available online at: https://ieeexplore.ieee.org/abstract/document/10387599 (accessed January 9, 2024).

[3] Luidold C Schaberreiter T Wieser C Koumpis A Cappiello C Citro T. Increasing cybersecurity awareness and collaboration in organisations and local / regional networks: the CS-AWARE-NEXT project. In: Sustainable, Secure, and Smart Collaboration (S3C) Workshop 2023. (2023). Available online at: http://eprints.cs.univie.ac.at/7835/ (accessed December 19, 2023).

[4] Argyris C. Double-loop learning and implementable validity. In:Tsoukas H Mylonopoulos N, editors. Organizations as Knowledge Systems: Knowledge, Learning and Dynamic Capabilities. London: Palgrave Macmillan UK (2004). p. 29–45.

[5] Lenders V Tanner A Blarer A. Gaining an edge in cyberspace with advanced situational awareness. IEEE Secur. Privacy. (2015) 13:65–74. 10.1109/MSP.2015.30

[6] Boyd J. Destruction and Creation. (1976). Available online at: https://www.semanticscholar.org/paper/Destruction-and-Creation-Boyd/483359fa9420efcddde5a17da597f462c2a788c2 (accessed December 15, 2023).

[7] Zager R Zager J. OODA loops in cyberspace: a new cyber-defense model. Small Wars J. (2017). Available online at: https://smallwarsjournal.com/jrnl/art/ooda-loops-cyberspace-new-cyberdefense-model

[8] Bayuk JL. Cyber security policy catalog. In: Cyber Security Policy Guidebook. Hoboken: John Wiley & Sons, Ltd. (2012). p. 93-210. Available online at: https://onlinelibrary.wiley.com/doi/abs/10.1002/9781118241530.ch6 (accessed December 19, 2023).

[9] Shaver KG. The Attribution of Blame: Causality, Responsibility, and Blameworthiness. Berlin: Springer Science & Business Media. (2012).

[10] Pala A Zhuang J. Information sharing in cybersecurity: a review. Deci Analy. (2019) 16:157–237. 10.1287/deca.2018.038719642375

[11] Pollini A Callari TC Tedeschi A Ruscio D Save L Chiarugi F. Leveraging human factors in cybersecurity: an integrated methodological approach. Cogn Technol Work. (2022) 24:371–90. 10.1007/s10111-021-00683-y 34149309 PMC8195225

[12] Langner G Andriessen J Quirchmayr G Furnell S Scarano V Tokola TJ. Poster: the need for a collaborative approach to cyber security education. In: 2021 IEEE European Symposium on Security and Privacy (EuroS&P). Vienna (2021). p. 719–721. 10.1109/EuroSP51992.2021.00058

